# Authorship decision-making in the field of orthopedic surgery and sports medicine

**DOI:** 10.1016/j.jcot.2021.101531

**Published:** 2021-07-28

**Authors:** F. Ruben H. Nurmohamed, Istifari Voigt, Preshant Awadpersad, Roshni H.S. Matawlie, Pravesh S. Gadjradj

**Affiliations:** a Faculty of Medicine, Utrecht University Medical Center, the Netherlands; b Department of Neurosurgery, Park MC, Rotterdam, the Netherlands

**Keywords:** ICMJE-Guidelines, Honorary authorship, Orthopedics, Honorary authorship, HA, the International Committee of Medical Journal Editor, ICMJE

## Abstract

**Purpose:**

To facilitate decision-making in authorship positions, the International Committee of Medical Journal Editor (ICMJE) developed a guideline that stipulates criteria authors should meet in order to merit authorship. Authors who did not meet these criteria and still enlisted as authors, are called ‘honorary’ authors. In this study, the prevalence and characteristics of honorary authorship (HA) is assessed in the field of Orthopedics and Sports Medicine.

**Methods:**

A survey was distributed among corresponding authors of articles published in 2019 in six Orthopedics-dedicated journals.

**Results:**

479 of the 1392 approached authors responded, leading to a response rate of 34.4%. 91.6% of the respondents were aware of the ICMJE guidelines, whereas 67.8% were aware of the issue of HA. Overall, the prevalence of guideline-based HA was 41.9%, while the prevalence of self-perceived HA was 14.7%. Having a senior member automatically enlisted as author on the departments, was associated with a higher rate of guideline-based HA (OR 5.03) and self-perceived HA (OR 3.31).

**Conclusions:**

The prevalence of HA in the field of Orthopedics and Sports Medicine is high, but comparable to other medical fields. Transparency in authorship decision-making is crucial to maintain liability in scientific articles.

## 1 Introduction

In present-day medicine, publishing articles in scientific journals is a popular manner to contribute new knowledge to the academic world. However, publications can also be used to measure academic success and accomplishments of individual researchers, making it a considerable parameter to assess scientific excellence.^12^ The importance of publications is also shown in the trend that institutions and residency programs incorporate the amount of publications as criterion in their application process.^14^

With these social and academic implications, authorship ensures credit which is desired by academics. To ensure the responsibility of authorship, the International Committee of Medical Journal Editor (ICMJE) developed a guideline that stipulates criteria authors should meet in order to merit authorship.^1^ The four criteria are:

1. “Substantial contributions to the conception or design of the work; or the acquisition, analysis, or interpretation of data for the work”;
2. “Drafting the work or revising it critically for important intellectual content”;
3. “Final approval of the version to be published”;
4. “Agreement to be accountable for all aspects of the work in ensuring that questions related to the accuracy or integrity of any part of the work are appropriately investigated and resolved.”

Authors who did not meet these criteria and are still enlisted as authors are termed ‘honorary’ authors. Having a senior academic as an honorary author could be tempting for the first author, as the reputation of the senior may help the publication chances of the scientific work. The other way around, the senior academic receives authorship credit without the effort of giving scientific contribution (1). The prevalence of honorary authorship (HA) in scientific publications ranges from 25% to 63% in different medical specialties.^3^^,^^4^^,^^6^^,^^7^^,^^9^^,^^15^ Nevertheless, there is a general consensus that HA or ‘gift authorship’ is a violation of scientific integrity.

Until now, the literature is scarce about the issue and prevalence of HA in orthopedic-related publications. In this study, the prevalence and characteristics of HA is assessed in six leading journals for Orthopedics and Sports Medicine.

## 2 Methods

According to the height of the impact factor in 2019, six journals in the field of Orthopedics and Sports Medicine were selected. These journals were the British Journal of Sports Medicine (BJSM), the American Journal of Sports Medicine (AJSM), the Journal of Bone and Joint Surgery American Volume (JBJS), Clinical Orthopedics and Related Research (CORR), The Bone and Joint Journal (TBJJ) and Osteoarthritis and Cartilage (OAC).

PubMed was screened for email-addresses of corresponding authors of all articles published in 2019. All articles with more than 1 author and available email-addresses were included. Exclusion criteria were editorials and other correspondence-related articles.

Based on the literature, a questionnaire was constructed using SurveyMonkey.^4^^,^^6^^,^^15^ The questionnaire consists of 22 questions, divided into four parts:

1. Demographics of respondents
2. Awareness of the ICMJE authorship guidelines
3. Honorary authorship
4. Decision-making of authorship.

The survey made a distinction between two forms of HA, namely guideline-based HA and self-perceived HA. Guideline-based HA is defined as coauthors performing tasks that, when performed, should not lead to authorship (nonauthorship task). These tasks included contributing illustrations, proofreading, technical editing etc. Self-perceived HA is defined as a coauthor that obtained authorship wrongfully according to the corresponding author. Surveys were distributed from March till May 2020. Each corresponding author received only one survey per journal.

### 2.1 Statistical analysis

Data was collected by SurveyMonkey, *X*2 tests were performed to assess possible association between variables and presence of guideline-based or self-perceived HA. If the univariable analysis resulted in a trending or statically significant association (P < 0.10 or <0.05), the variable was used in a multivariable logistic regression model. The regression model was used to obtain adjusted odds ratios (ORs) of the variables associated with guideline-based or self-perceived HA. A P value < 0.05 was considered to be statistically significant. All statistical analyses were performed using SPSS version [25.0].

## 3 Results

**Fig. 1:**
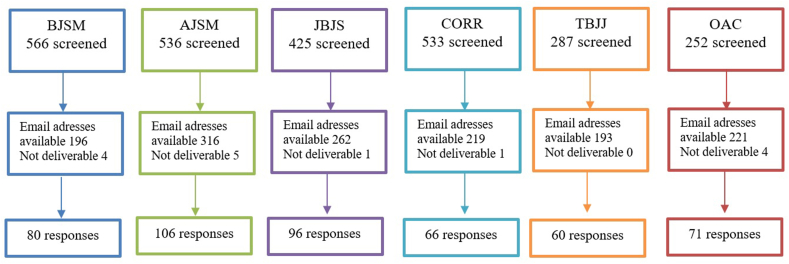
Flowchart of the study procedures.

For all articles published in 2019 in the selected journals, a total of 2599 articles were screened for eligibility. As shown in Fig. 1, 1392 corresponding authors had an available email-address for receiving an invitation. Eventually 479 of the eligible authors responded to the survey, leading to a response rate of 34.4%.

### 3.1 Demographics of respondents

**Fig. 2:**
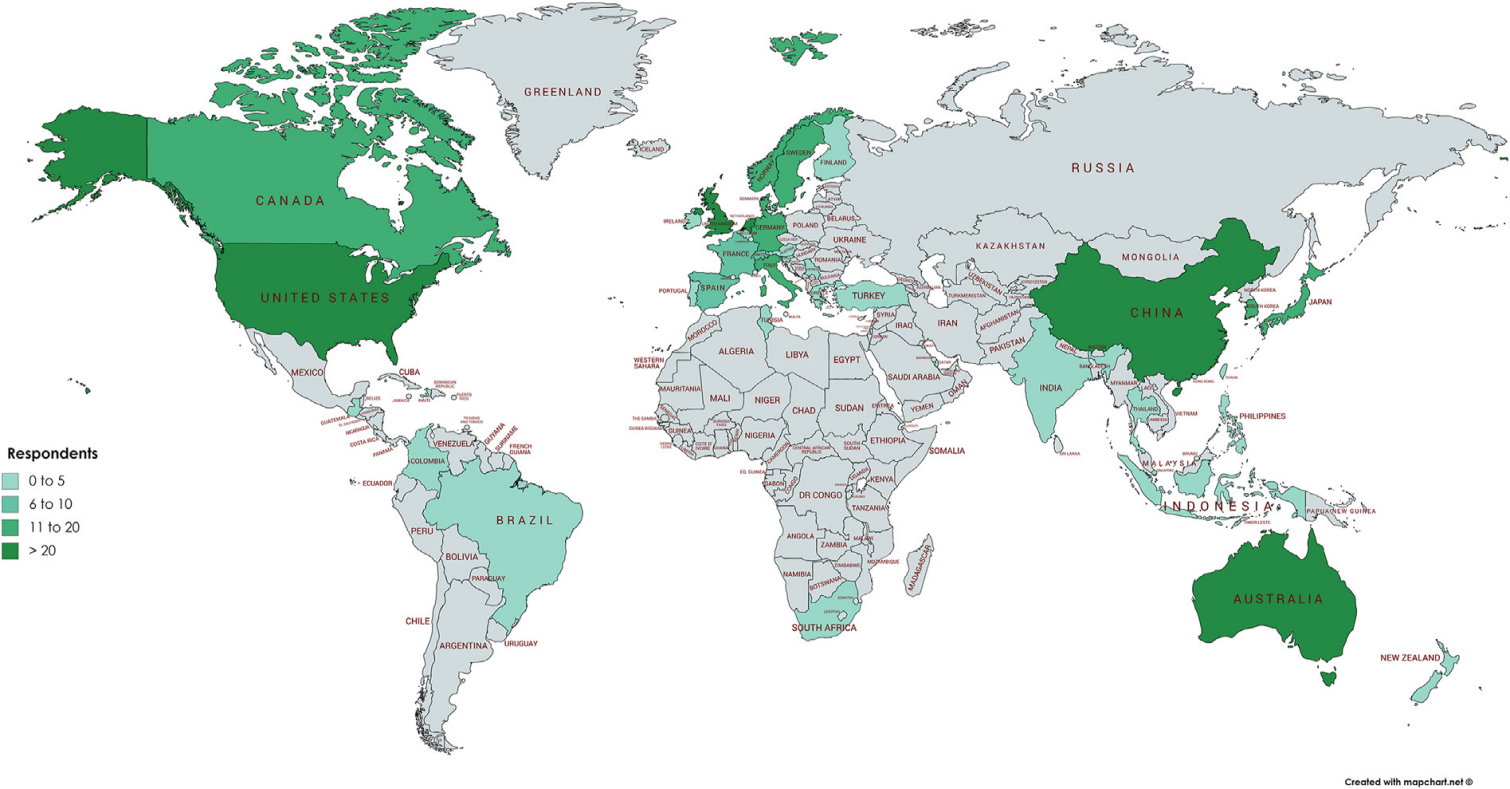
Map showing the respondents working locations.

Table 1 gives an overview of the demographics of responders. The vast majority was male, published 26 or more peer-reviewed articles, was employed as an Orthopedic surgeon and had 10 years or more professional experience either clinical-based or research-based. Furthermore, North America and Europe were mostly represented by the respondents with 75.1% (see Fig. 2).

**Table 1:** Demographics of respondents.

Characteristics	N (%)
Male	375 (78.3)
Number of peer-reviewed articles	
- <5	53 (11.1)
- 6-10	45 (9.4)
- 11-15	38 (7.9)
- 16-20	27 (5.6)
- 21-25	26 (5.4)
- >26	290 (60.5)
Primary profession	
- Orthopeadic surgeon	228 (47.6)
- PhD/Researcher	145 (30.6)
*Other MD*	43 (9.0)
- Sports medicine physician	28 (5.8)
- Paramedic	27 (5.6)
- Medical student	3 (0.6)
- Vetenary surgeon	1 (0.2)
- Research nurse	1 (0.2)
Lenght of professional experience	
- 1–2 years	23 (4.8)
- 3–5 years	78 (16.3)
- 6–10 years	89 (18.6)
- 10 years	289 (60.3)
Continent	
- North America	188 (39.2)
- Europe	172 (35.9)
- Asia	68 (14.2)
- Oceania	39 (8.1)
- South America	5 (1.0)
- Africa	5 (1.0)
- Central America	2 (0.4)

### 3.2 Awareness of the ICMJE authorship guidelines

Regarding authorship-guidelines, 91.6% (n = 439) of all invited authors were aware of the existence of the ICMJE-guidelines, whereas 67.8% (n = 325) knows of the general issue regarding HA (Table 2). In 12.7% (n = 61) of all articles, a senior member or department head was automatically listed as author. Of all respondents, 26.2% (n = 16) thought this was rarely or never justified, while 73.1% (n = 45) thought this was sometimes or always justified.

**Table 2:** Awareness of authorship guidelines and local agreements on authorship.

Awareness of	N (%)
- ICJME guidelines	439 (91.6)
- Guidelines of department or institution	86 (18.0)
- other authorship guidelines	21 (4.4)
- general issue honorary authorship	325 (67.8)
No awareness of authorship-related guidelines	25 (5.2)
Senior member of department, who is automatically enlisted as author on all manuscripts	61 (12.7)
Justified by corresponding author	
- never	6 (9.8)
- rarely	10 (16.4)
- sometimes	15 (24.6)
- most of the time	17 (27.2)
- always	13 (21.3)
Dispute regarding authorship order	199 (41.5%)
Professional relationship negatively influenced by dispute regarding authorship order	121 (25.3%)

Of all the respondents, 41.5% (n = 199) has been in a dispute regarding the authorship, which has had a negative influence on professional relationships among 25.3% (n = 121) of the authors.

### 3.3 Honorary authorship

**Fig. 3:**
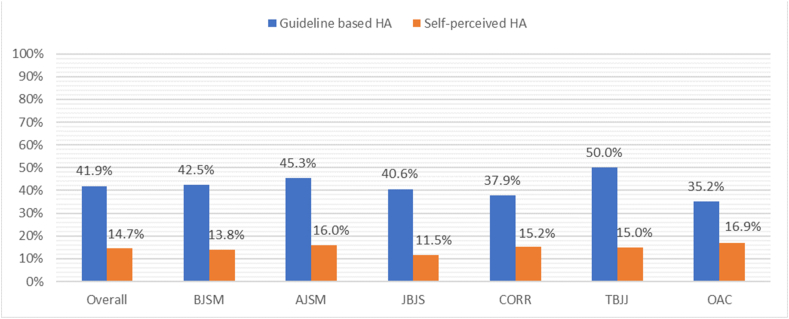
Prevalence Guideline based- and self-perceived HA.

An average of 41.9% (n = 201) of all invited authors indicated that one of their coauthors performed only tasks which should not result into authorship (guideline-based HA). The prevalence of guideline-based HA varied from 35.2% to 50.0% across the journals surveyed (Fig. 3). These non-authorship tasks were reviewing the manuscript in 34.4% (n = 165), approving the manuscript before submission to the journal in 29.9% (n = 143), signing the statement of copyright transfer to the journal in 20.9% (n = 100), obtaining funding or material support in 15.7% (n = 75), performing or treating the cases used in the study in 13.8% (n = 66), supervising or recruiting co-authors in 12.9% (n = 62), recruiting study subjects in 10.8% (n = 52) and contributing illustrations in 7.5% (n = 36).

Self-perceived HA was found in 14.7% (n = 70) among all survey respondents (Fig. 3). The prevalence of self-perceived HA varied from 11.5% to 16.9% across the journals surveyed.

Results of the multivariable logistic regression analyses (Table 3) showed that the presence of an automatically enlisted senior member was associated with a higher rate of self-perceived HA (OR 3.31, 95% CI 1.76 to 6.24) and a higher rate of ICMJE-defined HA (OR 5.03, 95% CI 2.89 to 9.78). Furthermore, a longer academic experience (≥10 years) of the corresponding author, was also associated with a higher rate of ICMJE-defined HA (OR 1.98, 95% CI 1.10 to 3.54).

**Table 3:** Multivariable logistic regression analysis on factors associated with self-perceived or ICMJE-defined honorary authorship.

		Self-perceived HA	ICMJE-defined HA
	*Reference*	*OR (95% CI)*	*OR (95% CI)*
Length of academic experience	<10 years	1.38 (0.92–2.08)	1.98 (1.10–3.54)∗
Aware of authorship guidelines	No	0.68 (0.33–1.41)	0.91 (0.34–2.44)
Aware of the general issue of HA	No	0.71 (0.45–1.10)	1.33 (0.67–2.64)
Presence of automatically listed senior member	No	3.31 (1.76–6.24)∗	5.03 (2.89–9.78)∗
Dispute regarding authorship order	No	1.18 (0.73–1.93)	1.70 (0.83–3.48)
Negatively affected relationship due to authorship decision	No	1.40 (0.80–2.46)	1.57 (0.74–3.36)
Senior member decided authorship order	No	1.39 (0.93–2.10)	1.67 (0.92–3.03)

∗p < 0.05.

### 3.4 Decision-making of authorship

Regarding the decision-making of authorship, in 47.8% (n = 229) of all studies, the authorship order was decided by the research group as whole. In 22.5% (n = 108) and 29.6% (n = 142), the authorship order was decided by the first or second author, respectively. The main criteria that was used to determine the order of the authors was in order of contribution (96.7%, n = 463). However, the last place in the authorship order was kept for a specific author. The author who provided the concept, supervision, and responsibility for all working steps of the project was placed last in 61.4% (n = 293). In 3.8% (n = 18), the author who is the most senior in the group but did not contribute to the study was placed last in the authorship order.

### 3.5 Opinions

**Fig. 4:**
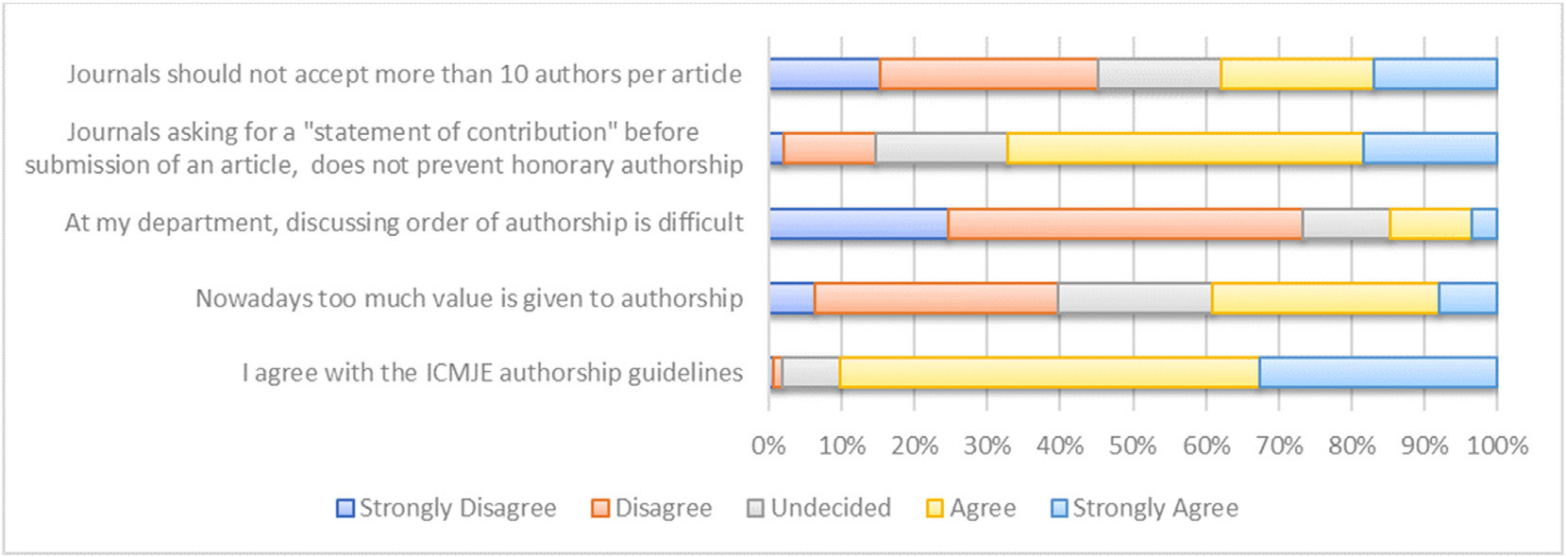
Opinions on five statements regarding authorship and authorship guidelines.

Fig. 4 gives an overview of the opinions of respondents regarding five statements. Majority of respondents (strongly) agreed with the ICMJE authorship guidelines (90.2%), but also (strongly) agreed that a ‘statement of contribution’ does not prevent HA (67.2%). Furthermore, 14.8% of the respondents (strongly) agrees that discussing the order of authorship is difficult at their department. Respondents were more divided on the statements that ‘nowadays too much value is given to authorship’ and that ‘journals should not accept more than 10 authors per article’.

## 4 Discussion

Honorary authorship is a phenomenon that exist across all medical specialties which can affect the integrity of the scientific literature. In this study, the presence of guidelines-based and self-perceived HA in the Orthopedic-related literature is assessed. The results show that the majority of authors (91.6%) is aware of the authorships guidelines.

Firstly, based on these ICMJE-guidelines, 41.9% (n = 201) of all listed authors performed non-authorship tasks. Only in 12.7% (n = 61) of all articles a senior member was automatically listed as author. Secondly, self-perceived HA was found in 14.7% of the articles, whereas 8.6% (n = 41) of the authors were asked to include an honorary author.

The presence of an automatically enlisted senior member was associated with a higher odds of ICMJE-based HA and self-perceived HA. In addition to that, having more academic experience was found to be associated with a higher rate of ICMJE-based HA.

Our findings are comparable with the prevalence's found in similar studies performed in scientific articles in other (bio)medical specialties. Previous studies in other medical fields such as Dermatology and Cardio-thoracic Surgery, found prevalences of ICMJE-based HA from 13.5% to 62.7%, while some other studies found prevalences of self-perceived HA of 13.5%–27.7%.^2^, ^3^, ^4^, ^5^, ^6^^,^^8^^,^^9^^,^^11^^,^^15^

Although, the considerable awareness of the ICMJE-guidelines, 4 out of 10 articles have listed authors with no basis. The ICMJE-guidelines are clear on what not authors’ tasks are, but with the ambiguity of the term “significant contribution” or the diminished attributed value which results in not applying the guidelines, could be an explanation of this discrepancy.

Enlisting a senior member increases the prevalence of self-perceived HA. This phenomenon could be explained by the culture that exists within the research department, where the senior member is enlisted out of respect. Another possibility could be that the corresponding author or research group list a senior member with authority to bestow prestige in the academic world and make their work more accessible.

Several limitations can be found in our study. First of all, the recall bias must be taken into account. The information collection was based on self-report of the corresponding authors. Although, our study is conducted roughly 5 months after the last publication, intervals between manuscript submission and publication and the specific tasks of corresponding author could influence the recall of authorship contributions or activities. Second, 34.4% (n = 479) of al contacted authors replied to our survey. Considering the response rate, response bias may be introduced. Third, only authors of six Orthopedics and Sports Medicine journals were contacted. One can argue that these journals have strict protocols for submitting manuscripts for authors. The prevalence of HA could be greater in journals with less strict protocols. In that case, our presented result would be an underestimation of the issue of HA.

In the literature, some solutions are proposed to tackle the issue of HA.^10^^,^^13^ Some of these proposed solutions are intended to establish a new authorship system e.g. by having a contributors system or a points-based system. Because overthrowing a whole authorship system can come with a lot of practical issues, other solutions seems more feasible. In a well-written paper by Smith et al., proposes a five-step best practice method to order authors in multi/interdisciplinary research.^13^ These steps included (1) to outline the roles and responsibilities; (2) determine the authorship order; (3) continuous dialogue on authorship contributorship and the order during the project; (4) making a final decision before submission of the manuscript and (5) draft a declaration on authorship and contributorship.

The phenomenon of honorary authorship has its effects on the integrity of scientific publications. It implicates that authors did not contribute to the scientific value of a study, while ensuring credit by obtaining authorship. And it may be the case that honorary authors can be held accountable for wrongdoing in publishing scientific articles.

Transparency in authorship decisions is crucial to maintain honesty and liability in scientific articles. Ways of determining author contributions should be developed to ensure that the problem of HA can be prevented where possible. The scientific community should be aware of the general issue of HA. Future research on methods of detecting HA should be performed to annul HA.

## Authors’ contributions

PG, RN, and IV conceived the study. PG and PA managed the study. IV, RM, and PA monitored and coordinated data collection. PG, RM, and IV designed the study. PG performed the analyses. All authors made substantial contributions to the design, data processing, and interpretation. RN drafted the article, and all other authors revised it critically for important intellectual content. PG is the guarantor. All authors had full access to all of the data in the study and can take responsibility for the integrity of the data and the accuracy of the data analysis. All authors read and approved the final manuscript.

## Funding

No financial support was received.

## Availability of data and materials

The datasets generated during and/or analyzed during the current study may be available from the corresponding author on reasonable request.

## Ethics approval and consent to participate

All research procedures were in accordance with the ethical standards of the 1964 Helsinki Declaration. Ethical approval was not required by the local IRB-board.

## Consent for publication

Not applicable.

## Declaration of competing interest

None.
